# Supplementary material for: Quantum advantage in postselected metrology
Source: Nat Commun. 2020 Jul 29;11:3775. doi: 10.1038/s41467-020-17559-w (PMC7391714; doi:10.1038/s41467-020-17559-w)
Supplement: Supplementary file 1 — Supplementary Information [file 41467_2020_17559_MOESM1_ESM.pdf]

Supplementary Information – Quantum Advantage in Postselected Metrology

David R. M. Arvidsson-Shukur, Nicole Yunger Halpern, Hugo V. Lepage, Aleksander A. Lasek, Crispin H. W. Barnes, and Seth Lloyd

(Dated: July 4, 2020)

SUPPLEMENTARY INFORMATION

Supplementary Note 1 – Expressing the postselected quantum Fisher information in terms of the KD distribution

As shown in the Results section of our main paper, the postselected quantum Fisher information is given by

$$\mathcal{I}_{\mathrm{Q}}(\theta|\Psi_\theta^{\mathrm{ps}}) = 4\langle\dot{\psi}_\theta^{\mathrm{ps}}|\dot{\psi}_\theta^{\mathrm{ps}}\rangle\frac{1}{p_\theta^{\mathrm{ps}}} - 4|\langle\dot{\psi}_\theta^{\mathrm{ps}}|\psi_\theta^{\mathrm{ps}}\rangle|^2\frac{1}{(p_\theta^{\mathrm{ps}})^2}, \tag{1}$$

where nonrenormalized postselected quantum state is $|\psi_\theta^{\mathrm{ps}}\rangle = \hat{F}\hat{U}(\theta)|\Psi_0\rangle$, where $|\Psi_0\rangle\langle\Psi_0| \equiv \hat{\rho}_0$. $p_\theta^{\mathrm{ps}} = \mathrm{Tr}(\hat{F}\hat{\rho}_\theta)$ is the probability of postselection.

In this supplementary note, we show that Supplementary Equation 1 can be expressed in terms of the doubly-extended KD distribution:

$$\mathcal{I}_{\mathrm{Q}}(\theta|\Psi_\theta^{\mathrm{ps}}) = 4\sum_{\substack{a,a',\\ f\in\mathcal{F}^{\mathrm{ps}}}}\frac{q_{a,a',f}^{\hat{\rho}_\theta}}{p_\theta^{\mathrm{ps}}}aa' - 4\Bigg|\sum_{\substack{a,a',\\ f\in\mathcal{F}^{\mathrm{ps}}}}\frac{q_{a,a',f}^{\hat{\rho}_\theta}}{p_\theta^{\mathrm{ps}}}a\Bigg|^2, \tag{2}$$

The first term of the quantum Fisher information (Supplementary Equation 1) is

$$\frac{4}{p_\theta^{\mathrm{ps}}}\langle\dot{\psi}_\theta^{\mathrm{ps}}|\dot{\psi}_\theta^{\mathrm{ps}}\rangle = \frac{4}{p_\theta^{\mathrm{ps}}}\mathrm{Tr}\big(\hat{F}\dot{\hat{U}}(\theta)\hat{\rho}_0\dot{\hat{U}}^\dagger(\theta)\hat{F}^\dagger\big) = \frac{4}{p_\theta^{\mathrm{ps}}}\mathrm{Tr}\Big(\hat{F}\hat{A}\hat{\rho}_\theta\hat{A}\Big) \tag{3}$$

$$= \frac{4}{p_\theta^{\mathrm{ps}}}\mathrm{Tr}\Big(\sum_a|a\rangle\langle a|a\hat{\rho}_\theta\sum_{a'}|a'\rangle\langle a'|a'\sum_{f\in\mathcal{F}_{\mathrm{ps}}}|f\rangle\langle f|\Big), \tag{4}$$

where, in Supplementary Equation 4, we have expressed $\hat{A}$ and $\hat{F}$ in their corresponding eigendecompositions. This expression can be rewritten in terms of the doubly extended Kirkwood-Dirac quasiprobability distribution ($q_{a,a',f}^{\hat{\rho}} = \langle f|a\rangle\langle a|\hat{\rho}|a'\rangle\langle a'|f\rangle$):

$$\frac{4}{p_\theta^{\mathrm{ps}}}\sum_{\substack{a,a',\\ f\in\mathcal{F}^{\mathrm{ps}}}}\mathrm{Tr}\left(aa'q_{a,a',f}^{\hat{\rho}_\theta}\frac{|a\rangle\langle f|}{\langle f|a\rangle}\right) = \frac{4}{p_\theta^{\mathrm{ps}}}\sum_{\substack{a,a',\\ f\in\mathcal{F}^{\mathrm{ps}}}}q_{a,a',f}^{\hat{\rho}_\theta}aa'. \tag{5}$$

Similarly, the second term of Supplementary Equation 1 is

$$\frac{4}{(p_\theta^{\mathrm{ps}})^2}\big|\langle\psi_\theta^{\mathrm{ps}}|\dot{\psi}_\theta^{\mathrm{ps}}\rangle\big|^2 = \frac{4}{(p_\theta^{\mathrm{ps}})^2}\big|\mathrm{Tr}\big(\hat{F}\hat{\rho}_\theta\hat{A}\big)\big|^2 = \frac{4}{(p_\theta^{\mathrm{ps}})^2}\Bigg|\sum_{\substack{a,a',\\ f\in\mathcal{F}^{\mathrm{ps}}}}q_{a,a',f}^{\hat{\rho}_\theta}a\Bigg|^2. \tag{6}$$

Combining the expressions above gives Supplementary Equation 2:

$$\mathcal{I}_{\mathrm{Q}}(\theta|\Psi_\theta^{\mathrm{ps}}) = 4\sum_{\substack{a,a',\\ f\in\mathcal{F}^{\mathrm{ps}}}}\frac{q_{a,a',f}^{\hat{\rho}_\theta}}{p_\theta^{\mathrm{ps}}}aa' - 4\Bigg|\sum_{\substack{a,a',\\ f\in\mathcal{F}^{\mathrm{ps}}}}\frac{q_{a,a',f}^{\hat{\rho}_\theta}}{p_\theta^{\mathrm{ps}}}a\Bigg|^2. \tag{7}$$

Supplementary Note 2 – Proof of Theorem 2

Here, we prove Theorem 2. First, we upper-bound the right-hand side of Supplementary Equation 2, assuming that all $q_{a,a',f}^{\hat{\rho}_\theta}/p_\theta^{\mathrm{ps}} \in [0,\,1]$. We label the M eigenvalues of $\hat{A}$ and arrange them in increasing order: $a_1, a_2, ..., a_M$, such that $a_1 \equiv a_{\min}$ and $a_M \equiv a_{\max}$. Initially, we assume that the 0-point of the eigenvalue axis is set such that $a_1 = 0$ and $a_M = \Delta a$. In this scenario, all the components of the first term of Supplementary Equation 2 are nonnegative. We temporarily ignore the form of $q_{a,a',f}^{\hat{\rho}_\theta}/p_\theta^{\mathrm{ps}}$, and treat this ratio as a general quasiprobability distribution. Then, $\mathcal{I}_{\mathrm{Q}}(\theta|\Psi_\theta^{\mathrm{ps}})$ maximizes when $q_{a,a',f}^{\hat{\rho}_\theta}/p_\theta^{\mathrm{ps}}$ vanishes at all a' values except $a' = a_{\max}$. We define $q_a \equiv \sum_{a',f\in\mathcal{F}^{\mathrm{ps}}} q_{a,a',f}^{\hat{\rho}_\theta}/p_\theta^{\mathrm{ps}}$,

such that all $q_a \in [0,\,1]$ and $\sum_a q_a = 1$. If $q^{\hat{\rho}_\theta}_{a,a',f}/p^{\mathrm{ps}}_\theta$ is nonzero only when $a' = a_{\max}$, Supplementary Equation 2 becomes

$$\mathcal{I}_{\mathrm{Q}}(\theta|\Psi^{\mathrm{ps}}_\theta) = 4a_M \sum_a q_a a - 4\Big(\sum_a q_a a\Big)^2. \tag{8}$$

Expanding each sum, we obtain

$$\begin{aligned}\mathcal{I}_{\mathrm{Q}}(\theta|\Psi^{\mathrm{ps}}_\theta) &= 4a_M(q_{a_1}a_1 + K + q_{a_M}a_M) - 4(q_{a_1}a_1 + K + q_{a_M}a_M)^2 && (9)\\ &= 4a_M(K + q_{a_M}a_M) - 4(K + q_{a_M}a_M)^2, && (10)\end{aligned}$$

where we used $q_{a_1}a_1 = 0$ and defined $K \equiv \sum_{a\in\{a_2,\ldots,a_{M-1}\}} q_a a \leq a_M$. As $\hat{A}$ is not totally degenerate, $a_M \neq 0$, and Supplementary Equation 10 is maximized when $q_{a_M} = (a_M - 2K)/(2a_M)$. This yields

$$\max\{\mathcal{I}_{\mathrm{Q}}(\theta|\Psi^{\mathrm{ps}}_\theta)\} = a_M^2 = (\Delta a)^2, \tag{11}$$

where we have recalled that $a_M = \Delta a$.

We are left with proving that we can always set $a_1 = 0$ and $a_M = \Delta a$. We continue to assume that $q^{\hat{\rho}_\theta}_{a,a',f}/p^{\mathrm{ps}}_\theta \in [0,\,1]$, and we shift all the eigenvalues by a constant real value δ_a. The effect on $\mathcal{I}_{\mathrm{Q}}(\theta|\Psi^{\mathrm{ps}}_\theta)$ is

$$\mathcal{I}_{\mathrm{Q}}(\theta|\Psi^{\mathrm{ps}}_\theta) \to 4 \sum_{\substack{a,a',\\ f\in\mathcal{F}^{\mathrm{ps}}}} \frac{q^{\hat{\rho}_\theta}_{a,a',f}}{p^{\mathrm{ps}}_\theta}(a+\delta_a)(a'+\delta_a) - 4\Big[\sum_{\substack{a,a',\\ f\in\mathcal{F}^{\mathrm{ps}}}} \frac{q^{\hat{\rho}_\theta}_{a,a',f}}{p^{\mathrm{ps}}_\theta}(a+\delta_a)\Big]^2 \tag{12}$$

$$= 4 \sum_{\substack{a,a',\\ f\in\mathcal{F}^{\mathrm{ps}}}} \frac{q^{\hat{\rho}_\theta}_{a,a',f}}{p^{\mathrm{ps}}_\theta} aa' - 4\Big[\sum_{\substack{a,a',\\ f\in\mathcal{F}^{\mathrm{ps}}}} \frac{q^{\hat{\rho}_\theta}_{a,a',f}}{p^{\mathrm{ps}}_\theta} a\Big]^2 + 4\delta_a\Big(\sum_{\substack{a,a',\\ f\in\mathcal{F}^{\mathrm{ps}}}} \frac{q^{\hat{\rho}_\theta}_{a,a',f}}{p^{\mathrm{ps}}_\theta} a - \sum_{\substack{a,a',\\ f\in\mathcal{F}^{\mathrm{ps}}}} \frac{q^{\hat{\rho}_\theta}_{a,a',f}}{p^{\mathrm{ps}}_\theta} a'\Big) = \mathcal{I}_{\mathrm{Q}}(\theta|\Psi^{\mathrm{ps}}_\theta). \tag{13}$$

The last equality holds because $q^{\hat{\rho}}_{a,a',f} = \big(q^{\hat{\rho}}_{a',a,f}\big)^*$ generally and we are assuming that $q^{\hat{\rho}}_{a,a',f} \in \mathbb{R}$. Consequently, if all $q^{\hat{\rho}_\theta}_{a,a',f}/p^{\mathrm{ps}}_\theta \in [0,\,1]$, then $\mathcal{I}_{\mathrm{Q}}(\theta|\Psi^{\mathrm{ps}}_\theta) \leq (\Delta a)^2$. The second term of Supplementary Equation 2 cannot be decreased by imaginary values in $q^{\hat{\rho}_\theta}_{a,a',f}$. Moreover, the first term is necessarily real and nonnegative. Thus imaginary elements $q^{\hat{\rho}_\theta}_{a,a',f}$ cannot increase $\mathcal{I}_{\mathrm{Q}}(\theta|\Psi^{\mathrm{ps}}_\theta)$. If $\mathcal{I}_{\mathrm{Q}}(\theta|\Psi^{\mathrm{ps}}_\theta) > (\Delta a)^2$, then $q^{\hat{\rho}_\theta}_{a,a',f}$ must have negative entries.

Supplementary Note 3 – Infinite postselected quantum Fisher information

Here, we show that the postselected quantum Fisher information $\mathcal{I}_{\mathrm{Q}}(\theta|\Psi^{\mathrm{ps}}_\theta)$ can approach infinity. The proof is by example; other examples might exist.

We assume that the generator $\hat{A}$ has $M \geq 3$ eigenvalues that are not all identical. We also assume that we possess an estimate θ_0 that lies close to the true value of θ: $\delta_\theta \equiv \theta - \theta_0$, with $|\delta_\theta| \ll 1$. (The derivation of the quantum Fisher information also rests on the assumption that one has access to such an estimate [1].)

By Supplementary Equations 1, 3 and 6,

$$\mathcal{I}_{\mathrm{Q}}(\theta|\Psi^{\mathrm{ps}}_\theta) = \frac{4}{p^{\mathrm{ps}}_\theta}\mathrm{Tr}\Big(\hat{F}\hat{A}\hat{U}(\theta)\hat{\rho}_0\hat{U}(\theta)^\dagger\hat{A}\Big) - \frac{4}{(p^{\mathrm{ps}}_\theta)^2}\Big|\mathrm{Tr}\Big(\hat{F}\hat{U}(\theta)\hat{\rho}_0\hat{U}(\theta)^\dagger\hat{A}\Big)\Big|^2. \tag{14}$$

We now choose $\hat{F}$ and $\hat{\rho}_0$ such that $\mathcal{I}_{\mathrm{Q}}(\theta|\Psi^{\mathrm{ps}}_\theta)$ approaches infinity. Crudely, p^{ps}_θ must approach 0 while $\mathrm{Tr}(\hat{F}\hat{A}\hat{U}(\theta)\hat{\rho}_0\hat{U}(\theta)^\dagger\hat{A})$ either stays constant or approaches 0 more slowly. We label the M eigenvalues of $\hat{A}$ and arrange them in increasing order: $a_1, a_2, ..., a_M$, such that $a_1 \equiv a_{\min}$ and $a_M \equiv a_{\max}$.

First, we choose $\hat{F} = |f_1\rangle\langle f_1| + |f_2\rangle\langle f_2|$, where

$$|f_1\rangle \equiv \frac{|a_{\max}\rangle + |a_{\min}\rangle}{\sqrt{2}}, \tag{15}$$

$$|f_2\rangle \equiv \frac{\frac{i}{\sqrt{2}}(|a_{\max}\rangle - |a_{\min}\rangle) + |a_k\rangle}{\sqrt{2}}, \tag{16}$$

and $|a_k\rangle \neq |a_{\max}\rangle$, $|a_{\min}\rangle$. We also choose $\hat{\rho}_0 = |\Psi_0\rangle\langle\Psi_0|$ such that

$$|\Psi_0\rangle \equiv |\Psi_0(\theta_0,\phi)\rangle = \hat{U}^\dagger(\theta_0)\frac{1}{\sqrt{2}}\Big\{[\cos(\phi)-\sin(\phi)]\frac{i}{\sqrt{2}}(|a_{\min}\rangle - |a_{\max}\rangle) + [\cos(\phi)+\sin(\phi)]\,|a_k\rangle\Big\}. \tag{17}$$

$\phi \approx 0$ is a parameter that can be tuned to maximize the postselected Fisher information for a given approximation accuracy δ_θ. As ϕ is a parameter of the input state, variations in the Fisher information with ϕ will reflect the effects of disturbances to the input state. Substituting the expressions for $\hat{F}$ and $\hat{\rho}_0$ into Supplementary Equation 14, we find

$$\begin{aligned}\mathcal{I}_{\mathrm{Q}}(\theta|\Psi_\theta^{\mathrm{ps}}) = {}& 8\big\{5 - 2\cos(2\phi)\big(\cos[(a_M-a_k)\delta_\theta] + \cos[(a_k-a_1)\delta_\theta]\big) + \cos[(a_M-a_1)\delta_\theta][\sin(2\phi)-1] - \sin(2\phi)\big\}^{-2} \\ & \times \Big\{2a_M^2 - a_M a_k + a_k^2 + 2a_1^2 - (3a_M+a_k)a_1 + (a_M-a_k)(a_k-a_1)\cos(4\phi)\big(\cos[(a_M-a_1)\delta_\theta]-1\big) \\ & + (a_M-a_k)(a_k-a_1)\cos[(a_M-a_1)\delta_\theta] + 2(a_M-a_1)\cos(2\phi)\big((a_1-a_k)\cos[(a_M-a_k)\delta_\theta] \\ & + (a_k-a_M)\cos[(a_k-a_1)\delta_\theta]\big) - 2(a_M-a_1)^2\sin(2\phi) + (a_M-a_1)\big((a_k-a_1)\cos[(a_M-a_k)\delta_\theta] \\ & + (a_M-a_k)\cos[(a_k-a_1)\delta_\theta]\big)\sin(4\phi)\Big\}. \end{aligned} \tag{18}$$

The postselection probability is

$$p_\theta^{\mathrm{ps}} = \frac{1}{8}\Big\{5 - 2\cos(2\phi)\big(\cos[(a_M-a_k)\delta_\theta] + \cos[(a_k-a_1)\delta_\theta]\big) + \cos[(a_M-a_1)\delta_\theta][\sin(2\phi)-1] - \sin(2\phi)\Big\}. \tag{19}$$

In the limit as our estimate θ_0 approaches the true value of θ, such that $\delta_\theta \to 0$,

$$\lim_{\delta_\theta\to 0} p_\theta^{\mathrm{ps}} = \sin^2(\phi), \tag{20}$$

$$\lim_{\delta_\theta\to 0} \mathcal{I}_{\mathrm{Q}}(\theta|\Psi_\theta^{\mathrm{ps}}) = \frac{(\cot(\phi)-1)^2}{2}(\Delta a)^2, \quad \text{and} \tag{21}$$

$$\lim_{\delta_\theta\to 0} p_\theta^{\mathrm{ps}} \times \mathcal{I}_{\mathrm{Q}}(\theta|\Psi_\theta^{\mathrm{ps}}) = \frac{1}{2}[1-\sin(2\phi)](\Delta a)^2. \tag{22}$$

In the limit as $\phi \to 0$,

$$\lim_{\phi\to 0}\Big[\lim_{\delta_\theta\to 0} p_\theta^{\mathrm{ps}}\Big] = 0, \tag{23}$$

$$\lim_{\phi\to 0}\Big[\lim_{\delta_\theta\to 0} \mathcal{I}_{\mathrm{Q}}(\theta|\Psi_\theta^{\mathrm{ps}})\Big] = \infty, \quad \text{and} \tag{24}$$

$$\lim_{\phi\to 0}\Big[\lim_{\delta_\theta\to 0} p_\theta^{\mathrm{ps}} \times \mathcal{I}_{\mathrm{Q}}(\theta|\Psi_\theta^{\mathrm{ps}})\Big] = \frac{1}{2}(\Delta a)^2. \tag{25}$$

According to Supplementary Equation 24, if first δ_θ and then ϕ approaches 0 in Supplementary Equation 18, $\mathcal{I}_{\mathrm{Q}}(\theta|\Psi_\theta^{\mathrm{ps}})$ approaches infinity.

There are a few points to note. First, $\mathcal{I}_{\mathrm{Q}}(\theta|\Psi_\theta^{\mathrm{ps}})$ diverges in the two ordered limits. In any real experiment, one could not blindly set $\phi = 0$, but would have to choose ϕ based on an estimate of θ. Second, if $\delta_\theta \approx 0$, then $\theta_0 \approx \theta$, and the pre-experiment variance of our initial estimate θ_0, $\mathrm{Var}(\theta_0)$, must be small. That is, we begin the experiment with much information about θ. Guided by the Cramér-Rao bound, we expect that, in a useful experiment, $\mathcal{I}_{\mathrm{Q}}(\theta|\Psi_\theta^{\mathrm{ps}})$ would grow large, while $1/\mathrm{Var}(\theta_0) < \mathcal{I}_{\mathrm{Q}}(\theta|\Psi_\theta^{\mathrm{ps}})$. Supplementary Figure 1 shows $\mathcal{I}_{\mathrm{Q}}(\theta|\Psi_\theta^{\mathrm{ps}}) \times \mathrm{Var}(\theta_0)$ as a function of ϕ and δ_θ for an experiment where $a_1 = -1$, $a_k = 1$, $a_M = 3$ and $\mathrm{Var}(\theta_0) = 10^{-6}$. If θ_0 is within a few $\sigma_{\theta_0} \equiv \sqrt{\mathrm{Var}(\theta_0)}$ of θ, then $\mathcal{I}_{\mathrm{Q}}(\theta|\Psi_\theta^{\mathrm{ps}}) \times \mathrm{Var}(\theta_0) \gg 1$. Supplementary Figure 1 shows that large values of $1/\delta_\theta$ can result in even larger values of $\mathcal{I}_{\mathrm{Q}}(\theta|\Psi_\theta^{\mathrm{ps}})$. Supplementary Figure 1 also illustrates the effect of input-state disturbances of ϕ on $\mathcal{I}_{\mathrm{Q}}(\theta|\Psi_\theta^{\mathrm{ps}}) \times \mathrm{Var}(\theta_0)$. Third, while the theoretical strategy investigated in this appendix achieves an infinite postselected quantum Fisher information, the postselection also "wastes" information as $\lim_{\phi\to 0}[\lim_{\delta_\theta\to 0} p_\theta^{\mathrm{ps}} \times \mathcal{I}_{\mathrm{Q}}(\theta|\Psi_\theta^{\mathrm{ps}})] < (\Delta a)^2$. If $\hat{A}$ possesses certain properties, it is possible to avoid wasting information through the postselection; we show how in the following appendix.

Supplementary Note 4 – Infinite postselected quantum Fisher information without loss of information

If the generator $\hat{A}$ has $M \geq 4$ eigenvalues, and the minimum and maximum eigenvalues are both at least doubly degenerate, then $\mathcal{I}_{\mathrm{Q}}(\theta|\Psi_\theta^{\mathrm{ps}})$ can approach infinity without information's being lost in the events discarded by postselection. We show how below.

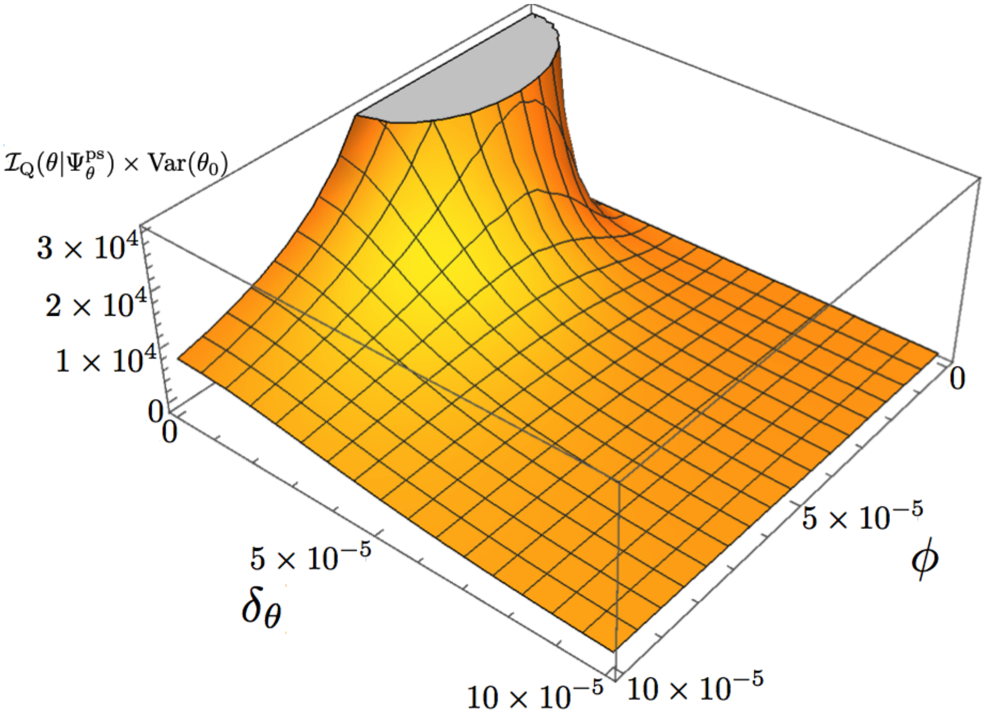

Supplementary Figure 1. **Scaled postselected quantum Fisher information.** The figure shows the postselected quantum Fisher information (Supplementary Equation 18) multiplied by the pre-experiment variance $\mathrm{Var}(\theta_0)$ as a function of ϕ and δ_θ. For small values of δ_θ and ϕ, the value of $\mathcal{I}_Q(\theta|\Psi_\theta^{ps}) \times \mathrm{Var}(\theta_0)$ diverges. The eigenvalues a_1, a_k and a_M are set to -1, 1 and 3, respectively. $\mathrm{Var}(\theta_0)$ was set to 1×10^{-6}.

First, we assign the orthonormal eigenvectors $|a_{\min_1}\rangle$ and $|a_{\min_2}\rangle$ to the eigenvalues $a_1 = a_{\min}$ and $a_2 = a_{\min}$, respectively. Here, we have reused the eigenvalue notation from Supp. Mat. . Similarly, we assign the orthonormal eigenvectors $|a_{\max_1}\rangle$ and $|a_{\max_2}\rangle$ to the eigenvalues $a_M = a_{\max}$ and $a_{M-1} = a_{\max}$, respectively. Second, we set $\hat{F} = |f_1\rangle\langle f_1| + |f_2\rangle\langle f_2|$, where

$$|f_1\rangle \equiv \frac{|a_{\max_2}\rangle - |a_{\min_1}\rangle}{\sqrt{2}}, \tag{26}$$

$$|f_2\rangle \equiv \frac{|a_{\min_2}\rangle - |a_{\max_1}\rangle}{\sqrt{2}}. \tag{27}$$

We also choose $|\Psi_0\rangle$ such that

$$|\Psi_0(\theta_0,\phi)\rangle = \hat{U}^\dagger(\theta_0)\frac{1}{2}\Big\{[\cos(\phi) - \sin(\phi)](|a_{\max_2}\rangle + |a_{\min_2}\rangle) + [\sin(\phi) + \cos(\phi)](|a_{\max_1}\rangle + |a_{\min_1}\rangle)\Big\}. \tag{28}$$

As in App. , $\phi \approx 0$ is a parameter that can be tuned to maximize $\mathcal{I}_Q(\theta|\Psi_\theta^{ps})$ for a given approximation accuracy of δ_θ.

Substituting the expressions for $\hat{F}$ and $\hat{\rho}_0$ into Supplementary Equation 14, we find

$$\mathcal{I}_Q(\theta|\Psi_\theta^{ps}) = \frac{\sin^2(2\phi)(a_M - a_1)^2}{\big(1 - \cos(2\phi)\cos[(a_M - a_1)\delta_\theta]\big)^2}. \tag{29}$$

The postselection probability is

$$p_\theta^{ps} = \frac{1}{2}\Big\{1 - \cos(2\phi)\cos[(a_M - a_1)\delta_\theta]\Big\}. \tag{30}$$

Again, we investigate the limit as our estimate θ_0 approaches the true value of θ:

$$\lim_{\delta_\theta \to 0} p_\theta^{ps} = \sin^2(\phi), \tag{31}$$

$$\lim_{\delta_\theta \to 0} \mathcal{I}_{\mathrm{Q}}(\theta|\Psi_\theta^{\mathrm{ps}}) = \cot^2(\phi)(\Delta a)^2, \text{ and} \tag{32}$$

$$\lim_{\delta_\theta \to 0} p_\theta^{\mathrm{ps}} \times \mathcal{I}_{\mathrm{Q}}(\theta|\Psi_\theta^{\mathrm{ps}}) = \cos^2(\phi)(\Delta a)^2. \tag{33}$$

In the limit as $\phi \to 0$,

$$\lim_{\phi \to 0} \left[\lim_{\delta_\theta \to 0} p_\theta^{\mathrm{ps}} \right] = 0, \tag{34}$$

$$\lim_{\phi \to 0} \left[\lim_{\delta_\theta \to 0} \mathcal{I}_{\mathrm{Q}}(\theta|\Psi_\theta^{\mathrm{ps}}) \right] = \infty, \text{ and} \tag{35}$$

$$\lim_{\phi \to 0} \left[\lim_{\delta_\theta \to 0} p_\theta^{\mathrm{ps}} \times \mathcal{I}_{\mathrm{Q}}(\theta|\Psi_\theta^{\mathrm{ps}}) \right] = (\Delta a)^2. \tag{36}$$

In conclusion, the above strategy allows us to obtain an infinite value for $\mathcal{I}_{\mathrm{Q}}(\theta|\Psi_\theta^{\mathrm{ps}})$, while $p_\theta^{\mathrm{ps}} \times \mathcal{I}_{\mathrm{Q}}(\theta|\Psi_\theta^{\mathrm{ps}}) = (\Delta a)^2$. No information is lost in the postselection. As in Supplementary Note 3, the results hold for the two ordered limits.

$$\mathcal{I}_{\mathrm{Q}}(\theta|\Psi_\theta^{\mathrm{ps}}) \times \mathrm{Var}(\theta_0)$$

Supplementary references

[1] Samuel L. Braunstein and Carlton M. Caves, "Statistical distance and the geometry of quantum states," Phys. Rev. Lett. **72**, 3439–3443 (1994).
